# Supplementary material for: A comparative ‘omics’ approach for prediction of candidate Strongyloides stercoralis diagnostic coproantigens
Source: PLoS Negl Trop Dis. 2023 Apr 17;17(4):e0010777. doi: 10.1371/journal.pntd.0010777 (PMC10138266; doi:10.1371/journal.pntd.0010777)
Supplement: S1 File — (DOCX) [file pntd.0010777.s001.docx]

S1. Number of outgroup protein hits for *S. stercoralis* protein families from the differentially expressed (DE) dataset.

| Part of Figure 4 | Protein family | *S. stercoralis* cluster in DE (n) | E-value | raw hits against 15 outgroups | unique hits against 15 outgroups | unique hits against *S. stercoralis* where relevant | Number of sequences in tree |
| --- | --- | --- | --- | --- | --- | --- | --- |
| A | SCP/TAPS | **19** | -10 | 5292 | **493** | n/a | 512 |
| B | TTL | **13** | -5 | 722 | **290** | n/a | 303 |
| C | AChE | **19** | -10 | 9209 | **576** | n/a | 595 |
| D | Aspartic peptidase | **2** | -5 | 850 | **282** | **9** | 293 |
| E | POP | **5** | -5 | 447 | **90** | **10** | 105 |
