# Supplementary material for: A comparative ‘omics’ approach for prediction of candidate Strongyloides stercoralis diagnostic coproantigens
Source: PLoS Negl Trop Dis. 2023 Apr 17;17(4):e0010777. doi: 10.1371/journal.pntd.0010777 (PMC10138266; doi:10.1371/journal.pntd.0010777)
Supplement: S6 File — FASTA headers are in the format: accession number, start aa, end aa. (DOCX) [file pntd.0010777.s006.docx]

**S6. Candidate coproantigen sequences in FASTA format**

FASTA headers are in the format: accession number, start aa, end aa.

# SCP/TAPS

### >SSTP_0000990000_363-743

KRKFLSLNYLNIRQRKVSNSKGVNCVTHVFEYYVRFMRGKYFMVFGGTTYPNEKDLIYDLIRQYPELKNQEVYLRGLGEITSTYMLYKVYEPAYAKVCLSDPYDKENKKSKVKGISDNEVIKHSSLKKVTFVCNKTTVPSLTHLAKCALYGQPSEVGANCERYFSKHDPPNCVEKVSFKLPNSKRCSNSEDVVLEALYSKLGTNKFTDSVWKAIWLSSINFACFSYKKYLLLKQRYLRELNSYRTAHEAPSLVESSELSKIAQSYAANLNQIKQKYRDDGKEFELIVDSTDFLTAPLLIKKWYEESSYYDYRFGRINSKCQNFAKLIWKNTAIIGIGVAKNSCKLNVVLLLYPKRRFASSNLWNIKKRRVSNLKRSRSLSE

# TTL

### >SSTP_0000700800_1-177

MVYAQGHITCDGEPLNDIVVKLINEKKKAFDVVKDTVRTHNDGRFYLQGDFKDFYKINPQVKFWHKCFVKSWNFFKNICYYEYTLLFNAEHCTRVEPKKPEKPKKSKKHRKSKRSKKHRKSKKSKKSKKSENSEKSENSEKSEEFEDFKPYLFCDYKEIKLKKNNVSGHKNCFAWGK

### > SSTP_0001222000_22-50

FKKKPSSKPKPTPKPKPIPIYISAYGHIT

### > SSTP_0001222000_51-86

CNGKDMKFITVTLLDVRQRKKTYVMGKKKTRRTGDF

### > SSTP_0001222000_65-159

DVRQRKKTYVMGKKKTRRTGDFFIRGKVKYPPNYEPKLKFTYKCHKKAPKKTYCLKKNDSKFPVVDEIKRKNDTIHIYFFNEIKVDDNFKNGMNK

### > SSTP_0001222000_87-116

FIRGKVKYPPNYEPKLKFTYKCHKKAPKKT

### > SSTP_0001222000_125-160

KFPVVDEIKRKNDTIHIYFFNEIKVDDNFKNGMNKC

# AChE

### > SSTP_0000274700_17-43

YDEPNITTYYGNITGTKITVLGQKMTE

### > SSTP_0000274700_63-104

REINKDHFKTTYHAVHLANSCPQIIRLMNFSGYNDSNPTNNI

### > SSTP_0000274700_187-207

QMVLKWINRTIESFNGNKSEV

### > SSTP_0000274700_243-266

THFMNTVSPIIAEINTLNVSVMVN

### > SSTP_0000274700_268-306

TKDTIFNKTTIRLDSKNLNYYYQNIINKEEKNNTAILEC

### > SSTP_0000274700_308-328

RTKNVSELLEAANKVRAKGQM

### > SSTP_0000274700_372-451

ATFFMATSFTNNTNLNCSFYPQLPTNDTKNQCDMTKKNFKHLVDYVATLLKFSKNETLKLRRIYNKYGSTYTNRTIRLLS

### > SSTP_0000274700_551-607

VSNPEAIVFNETLIKKSEPLYTNVELLTCKKLLKLIQQSKEKKQISKDLFNQLRPSN

### >SSTP_0000509400_85-103

PKEYNFSGLKSWYPEKHKM

### >SSTP_0000509400_390-411

MINEELTNQSFIPIRKLYNKFG

# Aspartic peptidase

### >SSTP_0000164500_107-140

GGSVDCPKYCSNAALCPFLCQLICCDKNKETKSN

# POP

### > SSTP_0001108800_221-266

KYLSDKLENIKLTNLAFAYNGKGFFYSTYGKKKDQNNPIDNGEYMH

### > SSTP_0001108800_384-421

KGEKVWKELIKTDSKRKIKSVTAAGGRYLIVNYLEDLE

### > SSTP_0001108800_422-507

DKVYIHDKWTGKMITKLDLEPGSIVSITASTHQSRFFIKVSSQVTPQTMYTGNLLELKHNKKINMKVIIKPIISGVKKSDYVIKKI

### > SSTP_0001108800_741-779

VSDARERNGVVTFRSNIDETIDIFAFIKETLNIKWKYDV
